# Body Dysmorphic Disorder in Plastic Surgery

**Published:** 2013-06-21

**Authors:** Kashyap K. Tadisina, Karan Chopra, Devinder P. Singh

**Affiliations:** Division of Plastic Surgery, University of Maryland Medical Center, Baltimore

**Figure:**
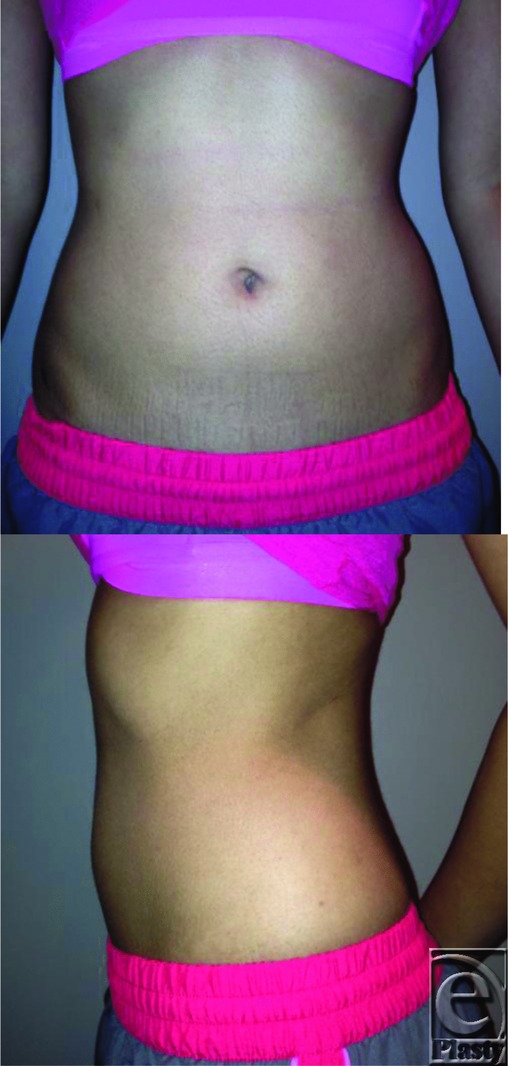


## DESCRIPTION

A 19-year-old college student presents to the plastic surgery clinic, seeking evaluation for abdominal liposuction. This is her fourth consultation seeking liposuction after being turned down by 3 other local plastic surgeons. Further questioning reveals that she spends 2 to 3 hours a day analyzing her body and that her perception of increased abdominal girth has caused her significant emotional distress, resulting in decreased social interaction with friends and family. Physical examination reveals a pleasant woman who is thin, with a weight of 52 kg, and 165 cm (body mass index = 19 kg/m^2^). Surgical intervention was deferred, and after counseling, the patient agreed not to pursue surgery at this time.

## QUESTIONS

1. **What is body dysmorphic disorder (BDD), and what are its identifying characteristics?**
2. **What are the risks associated with operating on a patient with BDD?**
3. **How does a surgeon screen for BDD?**
4. **How does the surgeon proceed if he or she suspects a patient has BDD?**

## DISCUSSION

Body dysmorphic disorder is a psychiatric condition defined by 3 characteristics: (1) an obsession or preoccupation with a minor or nonexistent flaw in physical appearance that (2) causes functional impairment or significant distress that (3) is not explained by another psychological disorder.^1^^-^4 The flaw or defect may involve the body as a whole or one particular area. Body dysmorphic disorder affects about 1% to 2% of the general population but has been found to be up to 15 times more prevalent in patients seeking plastic surgery.^1^^,^^2^^,^^4^ Gender distribution is roughly equal between men and women, and patients are usually young, with a mean age of onset of 16.4 years. Patients usually seek their first surgical consultation in their 30s.^1^ The course of BDD is gradual and chronic in nature and can be exacerbated by social stressors or even surgical intervention.^1^^-^4 The distinguishing symptom of BDD is significant body image dissatisfaction. Patients suffering from BDD also engage in obsessive-compulsive behaviors, including mirror gazing, comparing personal features, excessive camouflaging, skin picking, reassurance seeking, and even “self-surgery” practices.^1^^-^5^,^^6^ Patients with BDD often have poor insight and frequently seek plastic surgery consultation over psychiatric consultation. These patients also have an increased tendency to engage in violent or threatening behaviors toward their surgeon.^4^ Because of their habits, BDD patients often times have broken social relationships, live alone, and avoid social situations where their perceived defect will be noticed.^2^

Studies have shown BDD to have a neurobiological basis, characterized by distinct psychosocial habits and perceptual biases that surgery alone cannot remedy.^1^^,^^5^ Surgeons who choose to operate on patients with BDD are at an increased risk for litigation,^7^ potential violence, increased stress and frustration in serving this population, and low patient satisfaction after surgery.^2^^,^^4^

The first screening tool for a surgeon to identify BDD is obtaining a thorough history. The following signs can help the aesthetic plastic surgeon identify patients suffering from BDD: excessive requests for aesthetic surgery, dissatisfaction with previous surgeries, expectations that surgery will solve all of their problems, preoccupation with one defect, psychiatric history, unusual motivation for surgery, or demanding behaviors.^1^^-^4^,^^6^ Other tools at the disposal of surgeons are structured questionnaires exploring motivation for surgery or evaluation by a mental health professional before any intervention.

Once screened, if a surgeon suspects a patient has BDD, the proper course of action is to refer the patient to follow up with a psychiatrist. Mental health professionals such as psychologists or psychiatrists can serve as a safety and screening measure to help identify potentially dangerous patients with BDD.^4^ Although most literature discourages surgical intervention for patients diagnosed with BDD, the decision to operate on a patient with BDD is ultimately at the discretion of the plastic surgeon. On the basis of the patient's history, severity of symptoms, the procedure and defect under consideration, predicted satisfaction, and most importantly, patient safety and surgeon comfort level, surgeons may decide whether or not to operate.^2^^,^^3^ Plastic surgery offers patients not only an opportunity for functional restoration or aesthetic physical rejuvenation, but many believe there are mental benefits as well,^4^^,^^5^^,^^8^ and surgery has even been discussed as a potential treatment for patients with BDD in conjunction with psychotherapy—a process defined as eumorphic plastic surgery.^8^^,^^9^
